# High Susceptibility of *Bt* Maize to Aphids Enhances the Performance of Parasitoids of Lepidopteran Pests

**DOI:** 10.1371/journal.pone.0000600

**Published:** 2007-07-11

**Authors:** Cristina A. Faria, Felix L. Wäckers, Jeremy Pritchard, David A. Barrett, Ted C.J. Turlings

**Affiliations:** 1 University of Neuchâtel, Institute of Zoology, E-vol, Neuchâtel, Switzerland; 2 University of Lancaster, The Lancaster Environmental Centre, Lancaster, United Kingdom; 3 University of Birmingham, School of Biosciences, Edgbaston, Birmingham, United Kingdom; 4 University of Nottingham, School of Pharmacy, Centre for Analytical Bioscience, Nottingham, United Kingdom; Cairo University, Egypt

## Abstract

Concerns about possible undesired environmental effects of transgenic crops have prompted numerous evaluations of such crops. So-called Bt crops receive particular attention because they carry bacteria-derived genes coding for insecticidal proteins that might negatively affect non-target arthropods. Here we show a remarkable positive effect of Bt maize on the performance of the corn leaf aphid *Rhopalosiphum maidis*, which in turn enhanced the performance of parasitic wasps that feed on aphid honeydew. Within five out of six pairs that were evaluated, transgenic maize lines were significantly more susceptible to aphids than their near-isogenic equivalents, with the remaining pair being equally susceptible. The aphids feed from the phloem sieve element content and analyses of this sap in selected maize lines revealed marginally, but significantly higher amino acid levels in *Bt* maize, which might partially explain the observed increased aphid performance. Larger colony densities of aphids on Bt plants resulted in an increased production of honeydew that can be used as food by beneficial insects. Indeed, *Cotesia marginiventris*, a parasitoid of lepidopteran pests, lived longer and parasitized more pest caterpillars in the presence of aphid-infested Bt maize than in the presence of aphid-infested isogenic maize. Hence, depending on aphid pest thresholds, the observed increased susceptibility of Bt maize to aphids may be either a welcome or an undesirable side effect.

## Introduction

With the rapid expansion of the commercial use of genetically modified (GM) plants, there is an increasing demand for information on their possible impact on non-target organisms. Of particular interests are parasitoids and predators that have an important function in pest regulation. To date several studies on the direct and indirect impact of GM plants on these beneficial insects have been conducted (reviewed by [1]–[3]), whereby most emphasis has been on so-called Bt plants, which are crops into which a gene has been incorporated from the entomopathogenic bacterium *Bacillus thuringiensis*. The introduced genes encode for the production of specific insecticidal proteins. An impact on entomophagous insects resulting from this transformation could be due to direct effects of the toxin, indirect effects via reduction in host or prey quantity and quality, or through unintended changes in plant characteristics caused by the insertion of the transgene. The first two potential effects have been widely investigated [1]–[3], but very few studies have specifically looked at the impact of other plant characteristics that may have unintentionally been altered as a result of transformation.

The primary targets of the Bt toxin are insects belonging to the Lepidoptera, Diptera and Coleoptera [4], [5]. This, and the fact that the toxin is not transported in the phloem [6]–[8] makes that aphids are very unlikely to be directly affected by the toxin. Recent reports suggest that aphids actually perform better on Bt maize lines than on their near isogenic counterparts [8]–[11], but the generality and cause of the differences remain, as yet, unknown. We too found indications that the corn leaf aphid, *Rhopalosiphum maidis* (Fitch) (Hemiptera: Aphididae), does better on Bt maize (unpubl.). These findings prompted the current study that aims to assess possible effects of the incorporation of the Bt gene into maize on the corn leaf aphid *R. maidis*, and to test if such effects reflect on the performance of *Cotesia marginiventris* (Cresson) (Hymenoptera: Braconidae), a generalist larval parasitoid of several important lepidopteran pests that can use aphid honeydew as a food source (Video S1) [12]. By including six distinct Bt lines in the study we could rule out that the consistent differences in aphid susceptibility between the transformed and near isogenic lines resulted from accidental changes due to differences in breeding history after transformation.

The six Bt lines, which covered three different transformation events, were indeed found to be significantly more susceptible to *R. maidis*. Subsequent analyses of phloem samples of transgenic and near-isogenic pairs were performed to determine if amino acid composition might explain the observed higher aphid performance on Bt maize.

As a consequence of the higher aphid numbers there were larger quantities of honeydew on Bt maize plants. Honeydew is often exploited as food by animals like honeybees, wasps, insect predators and even vertebrates [13]–[15]. It can also be a key alternative food source for parasitoids in the absence of plant-provided nectar [16]–[18], which is often the case in agricultural monocultures. We tested if the parasitic wasp *C. marginventris* might benefit from enhanced performance of aphids on Bt maize by measuring their longevity and parasitism rates in cages with aphid-infested transgenic maize and in cages with aphid-infested non-transformed isolines. Sugar composition and the intake by the wasps of honeydew from aphids on transgenic and isogenic plants were measured to reveal a possible explanation for the observed enhanced parasitoid performance on Bt maize.

## Materials and Methods

### Plants

All plants were individually grown from seed in a climate chamber (27±2°C, 60% r.h., 16L∶8D, and 50000 lm/m^2^). For measurements of aphid performance, six pairs of hybrids from one of the three commercially available Bt maize events and the correspondent near-isogenic lines were used: Bt11 (N4640Bt/N4640), Mon 810 (MEB 307Bt/Monumental, TXP138/EXP138, Novelis/Nobilis) and Event 176 (Valmont/Prelude, Navaris/Antaris). For the other experiments (honeydew analysis, parasitoid performance and amino acid composition of the phloem), only one pair of each of the three events was used: Bt11 (N4640Bt/N4640), Mon 810 (MEB 307Bt/Monumental) and Event 176 (Valmont/Prelude). All transgenic plants used express the *B. thuringiensis* gene which codes for the Cry1Ab toxin. The conventional lines used for comparison were Delprim, Pactol, Challenger, Byzance, Graf and Best. Barley of the variety Lyric was used for initial aphid rearing.

### Insects

The corn leaf aphid *R. maidis* was used in this study because it excretes copious amount of honeydew and despite of the fact that its pest status varies in different parts of the world, they usually do not cause economical damage to the crop [19]–[21]. The aphids were provided by the Agroscope RAC Changins in Switzerland and were reared in climate chambers (25°C, 70% r.h. and 14L∶10D) on barley unless otherwise specified.

*Spodoptera littoralis* (Boisduval) (Lepidoptera: Noctuidae) eggs were received weekly from Syngenta (Stein, Switzerland) and once the eggs hatched, the larvae were used for parasitoid rearing or in experiments. The *C. marginiventris* colony was maintained on *S. littoralis* larvae fed with artificial wheat germ based diet. Adults were kept in plastic Bugdorm-1 cages (30×30×30 cm, Megaview, Taiwan) at a sex ratio of 1∶2 (male:female) in climate chambers (25°C, 85% r.h. and 14L∶10D). Moist cotton wool was added to the cages to provide humidity and water for the wasps. The females used for the experiments were one day old, mated and unfed.

### Aphid performance

For these experiments aphids had been reared at least for four generations on the respective variety to avoid maternal effects. All plants were five weeks old at the start of an experiments, at which time they were infested with the aphids and transferred to climate chambers (25°C, 70% r.h. and 14L∶10D).

Mean relative growth rate MRGR [22] was used to measure the performance of individual aphids on the different varieties. For this, nymphs were individually weighed (initial weight: 50±10 µg) on a precision scale (Mettler MX5; ±2 µg) and placed in clipcages (1.5×1.5 cm) that were attached to the 6th and 8th leaves of the maize plants. Four days later the aphids were removed from the clipcages and weighed again. Each plant had two clipcages and there were 15 plants from each variety. The few aphids that disappeared were replaced by new ones. Differences in MRGR [(ln initial weight-ln final weight)/number of days] within each transgenic and near isogenic pairs were compared using the Mann-Whitney test.

To measure aphid colony performance a group of 100 *R.maidis* individuals (50 adults and 50 nymphs of mixed ages) were placed in clipcages attached to the 6th leaf of the maize plants. Three days later, when the aphids had settled on the plant, clipcages were removed and plants enclosed in sleeve cages (Megaview, Taiwan; 30×70 cm). Five weeks after infestation, the stem of each plant was cut close to the soil and the whole plant in the sleeve cage was put in a plastic bag and a beaker with ether was added to kill the aphids. Once the aphids were dead, they were removed from the plant with a brush and conserved in 70% ethanol. The aphids in ethanol were then put in a Petri dish of known area and the number of aphids present in 5% of the area of the Petri dish was counted. The total number of aphids on each plant was then estimated. Differences in the number of aphids were compared within each transgenic and near isogenic pair using the t-test.

### Stylectomy and amino acid analysis

A maximum of ten aphids were put overnight in a clip cage (1.5 cm of diameter) that was attached on a maize plant (three to four weeks old) overnight. Stylectomy was performed on the following day using high-frequency microcautery [23]. When a successful cut had been made, the exuding phloem sap was immediately collected into a water filled microcapillary. The sample volume was estimated by measuring the diameter of the sap droplet formed on the stylet after one minute of exudation and this measure was multiplied by the duration of the exudation. Sap was collected for a maximum of 90 minutes. After collection the samples were stored at −20°C. Once all samples were collected, they were transferred from the microcapillaries to Eppendorf tubes and placed in a desiccator so that the water in the samples would evaporate. The Eppendorf tubes were then stored at −20°C.

The amino acids were analysed by capillary electrophoresis with a Beckman P/ACE MDQ system equipped with a 488 nm argon-ion laser module (Picometrics, France, 25mW). The data was collected and analysed by Beckman P/ACE MDQ 1.5 or 1.2 software (Beckman-Coulter, Fullerton, CA, USA). Half an hour before analysis, the phloem samples were put at room temperature. For the analysis, 15–45 µl of the Dissolving Matrix (Sodium phosphate monobasis, Sodium phosphate dibasis, Glycine-Glycine) were added to the sample. Thereafter, the sample was mixed with 2.5–7.5 µl 50 mM NBD-F, and heated at 60°C for 3 min, and finally mixed with 15–45 µl DOPAC to quench the reaction and cooled down at room temperature before analysis. During capillary electrophoresis, the sample was injected by pressure at 0.5 psi for 5 s. The applied voltage for CE separation was 20.6 kV (0–16 min) and 30 kV (17–25min). CE experiments were conducted at 20°C [24].

The standard amino acid solution used for comparison contained 19 amino acids (L-Arginine, L-Alanine, L-Asparagine, L-Aspartic Acid, L-Glutamic Acid, L-Glutamine, Glycine, L-Histidine. L-Isoleucine, L-Leucine, L-Lysine, L-Methionine, L-Phenylalanine, L-Proline, L-Serine, L-Threonine, L-Tyrosine, L-Valine, L-Ornithine). The variation in amino acid composition of the transgenic and isogenic lines of each pair was statistically compared with separate t-tests for each amino acid and for the total amino acid concentration with the package SPSS 12.0 (SPSS Inc., Chicago). Additionally the distribution of amino acid concentrations was investigated by redundancy analysis (RDA), a direct gradient analysis, to explore the underlying trends in the dataset [25]. For these statistical analyses the data (amino acid concentrations of each sample) were log-transformed.

A principal component analysis (PCA) on the amino acid data was used to explore the relationship between aphid performance and amino acid concentration in the six maize varieties. The coordinates of the six varieties on the first principal axis were used as a composite variable, which expresses the most possible variation in the amino acid data. Values of the concentrations of amino acids were standardized prior to statistical analysis. Coordinates were used as independent variable in a linear regression to explain aphid performance. RDA and PCA analysis were conducted using the program CANOCO 4.5 (Biometrics, Wageningen).

### Parasitoid performance

Groups of three *C. marginiventris* females were placed in cages (50×50×100 cm) with one maize plant (5–6 weeks old) from a Bt variety or the corresponding non-transgenic line, these pairs comprised events Bt11 (N4640Bt/N4640), Mon 810 (MEB 307Bt/Monumental) or Event 176 (Valmont/Prelude). Two weeks prior to the tests, when plants were four weeks old, all plants were infested with around 400 *R. maidis* of mixed ages. All cages also contained two maize plants (three to four weeks old) of the conventional variety Delprim infested with around 150 *S. littoralis* larva (three to four days old) each.

The cages were sprayed with water twice per day. Mortality of the females was recorded daily and the *S. littoralis*-infested maize replaced every other day. The caterpillars from the replaced plants were collected and reared further on artificial diet until emerging parasitoids had formed cocoons, which were then counted. Differences in parasitism rate (numbers of cocoons formed) by *C. marginiventris* females kept in cages with different food sources were determined by a one-way ANOVA and differences between means compared using the Tukey's test with SPSS 12.0. In addition, effects of feeding on the honeydew from different maize varieties on survival probability of *C. marginiventris* were compared using survival analysis. Differences between survival curves of wasps feeding on honeydew from each pair of transgenic and near isogenic line were analysed with a log-rank test using S-Plus 6.2 (Insightful Inc., Seattle).

### Honeydew collection and analysis

All plants used for the honeydew collection were five to six weeks old and infested with around 200 *R. maidis* of mixed ages. Aphids were placed in clipcages on the 6th to the 8th leaf and one week later each clipcage was replaced by a new one, which was left on the plant for 24 h. After this period, these new clipcages were removed and then placed at 100% r.h. for 24 h and a micro-capillary was used to collect 1 µL of honeydew, which was diluted in 50 µL of 70% ethanol.

Just before analysing the samples they were diluted a further 1000× with Milli-Q water. Of each diluted sample, 10 µl was injected into a Dionex DX 500 HPLC-system (Dionex Corp., Sunnyvale, CA). The system was equipped with a GP 40 gradient pump, a Carbopac PA1 guard column (4×50 mm), a Dionex Carbopac PA1 analytical column (4×250 mm), as well as an ED 40 Electrochemical Detector for Pulsed Amperimetric Detection (PAD). The column was eluted with 1 M NaOH and Milli-Q water (10∶90%, 1 ml min-1) and kept at 20°C during analysis. Daily reference curves were obtained for sorbitol, mannitol, trehalose, glucose, fructose, melibiose, sucrose, melezitose, raffinose, maltose and erlose by injecting calibration standards with concentrations of 2.5 ppm, 5 ppm, 7.5 ppm, and 10 ppm of these sugars. The concentrations of the individual sugars were analysed using the program PEAKNET Software Release 5.1 (DX-LAN module).

### Correlation between honeydew intake and survival

As the sugar composition was similar among the different honeydews, we tested for differences in honeydew intake as an alternative explanation for the observed differences in parasitoid performance. For this we measured the honeydew intake of *C. marginiventris* females after one single feeding bout, and determined its effect on parasitoid survival.

*C. marginiventris* females were used when 24–30 h old. To make sure that the food intake was only due to sugar need, water was provided *ad libitum*. Consumption was determined by weighting the individual females on a precision scale (Mettler MX5; ±2 µg) before and immediately after exposure to honeydew. After this the females were kept individually in vials with moist cotton and their longevity was accessed daily.

Differences in the percentage of weight gained by *C. marginiventris* after one feeding bout on honeydew produced from the transgenic and near isogenic lines of each pair were compared within a pair using the t-test. Longevity was compared using the Mann-Whitney test. The correlation between honeydew intake and survival was determined by linear regression analysis.

## Results

### Aphid performance

**Figure 1:**
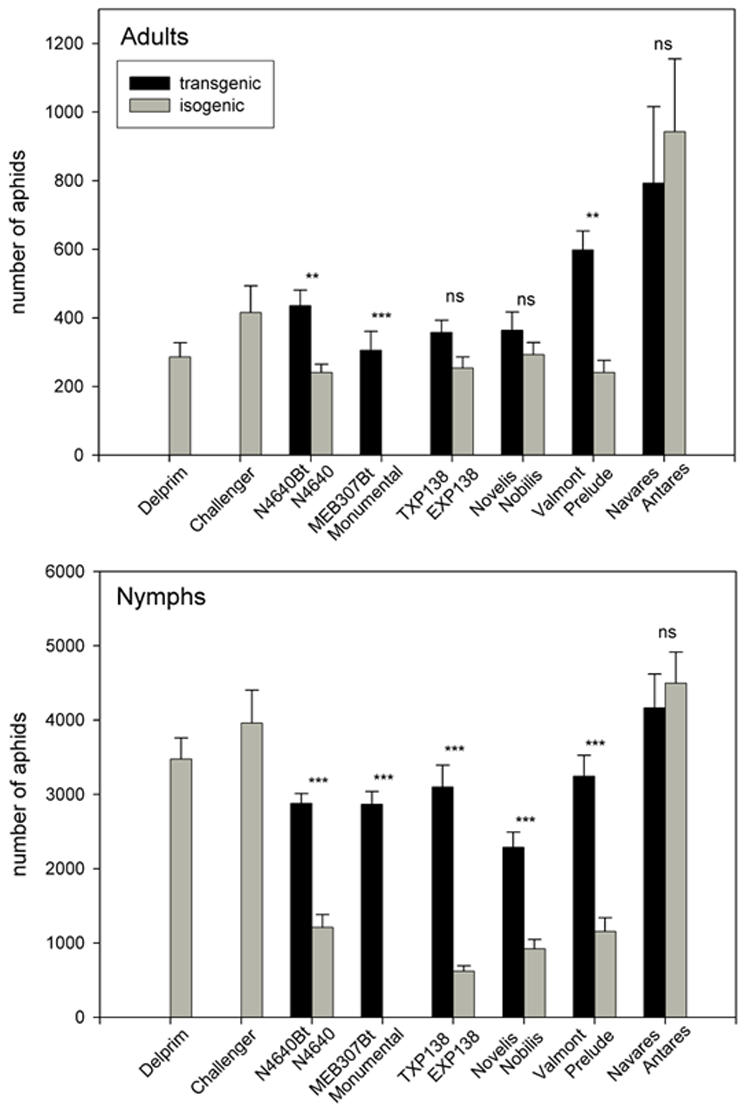
Aphid performance. Average number of *R. maidis* adults and nymphs on six pairs of transgenic and near isogenic varieties and on two conventional varieties (+SE). All comparisons are performed within each transgenic and near isogenic pair. Symbols indicate significant differences within each transgenic and isogenic pair (** p<0.01, *** p<0.001).

Aphid performance was compared for six pairs of transgenic and near isogenic lines belonging to three transformation events. Performance was measured at the individual, as well as population level and was also tested for two conventional varieties Delprim and Challenger. There were no differences in the mean relative growth rate (MRGR) of individual *R. maidis* within each of six pairs of transgenic and near isogenic lines tested (T = 212, p = 0.407 for N4640Bt/N4640; T = 258.5, p = 0.290 for TXP138/EXP138; T = 223.5, p = 0.724 for Novelis/Nobilis; T = 255, p = 0.351 for Valmont/Prelude; T = 253, p = 0.395 for Navares/Antares), except for the pair MEB 307Bt/Monumental, where the aphids did not survive on the near isogenic line (T = 345, p<0.001). These results contrast strongly with the results for colony performance (Figure 1). For all pairs, except Navares/Antares, there were significantly more nymphs on the transgenic lines than on the respective near isogenic lines (t = 7.745, df = 6, p<0.001 for N4640Bt/N4640; T = 26, p = 0.02 for MEB307Bt/Monumental; t = 8.216, df = 6, p<0.001 for TXP138/EXP138; t = 5.737, df = 6, p<0.001 for Novelis/Nobilis; t = 6.198, df = 6, p<0.001 for Valmont/Prelude; t = −0.533, df = 6, p<0.001 for Navares/Antares). For adults this was only the case for the pairs N4640Bt/N4640 and Valmont/Prelude (t = 3.770, df = 6, p<0.009 for N4640Bt/N4640; t = 5.501, df = 6, p = 0.002 for MEB307Bt/Monumental; t = 2.151, df = 6, p = 0.075 for TXP138/EXP138; t = 1.117, df = 6, p = 0.307 for Novelis/Nobilis; t = 5.437, df = 6, p = 0.002 for Valmont/Prelude; t = −0.485, df = 6, p = 0.645 for Navares/Antares). No aphids survived on the variety Monumental.

### Stylectomy and amino acid analysis

In order to investigate if the enhanced aphid performance on Bt maize resulted from differences in the amino acid composition of the phloem, we collected the phloem sieve element sap from three pairs of transgenic/near isogenic lines belonging to the events Bt11 (N4640Bt/N4640), Mon810 (MEB 307Bt/Monumental) and Event 176 (Valmont/Prelude). To exclusively obtain the sap that aphids normally ingest, it was collected directly from aphids stylets that were cut using high-frequency microcautery [26]. For this experiment we used the aphid *Rhopalosiphum padi* because this species is easier to handle for stylectomy procedure than *R. maidis*.

**Figure 2:**
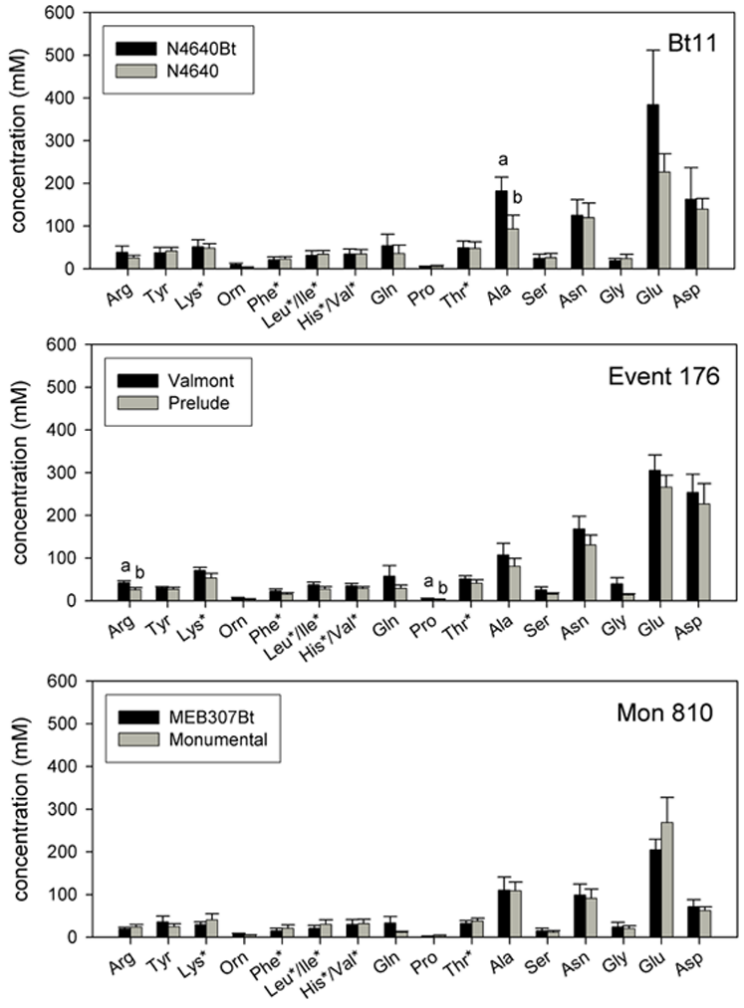
Phloem amino acid content. Amino acid concentrations (mM) in phloem samples from three transgenic varieties belonging to three transformation events and their correspondent near isogenic lines. Different letters indicate significant differences between amino acid concentration within one transgenic/isogenic pair (p<0.05). Amino acid abbreviations: arg, arginine; tyr, tyrosine; lys, lysine; orn, ornithine; phe, phenylalanine; leu/ile, leucine/isoleucine; his/val, histidine/valine; gln, glutamine; pro, proline; thr, threonine; ala, alanine; ser, serine; asn, asparagine; gly, glycine; glu, glutamate; asp, aspartate. Asterisks indicate the essential amino acids.

Eighteen amino acids were detected in sap samples: arginine, tyrosine, lysine, ornithine, phenylalanine, leucine, isoleucine, histidine, valine, glutamine, proline, threonine, alanine, serine, asparagine, glycine, glutamate, aspartate. Methionine was present in the standards that were used for identification, but was not found in any of the samples. However, the method is not suitable to detect small (<20 mM) amounts of methionine, so minor quantities of this amino acid in the sap may have gone undetected. All samples were dominated (81–87%) by non-essential amino acids. Two of the three transgenic/isogenic pairs showed significant differences in amino acid concentrations (Figure 2). For the pair N4640Bt/N4640, the concentration of the amino acid alanine was higher in the transgenic than in the isogenic variety (t = 2.609, df = 12, p = 0.02). For the pair Valmont/Prelude, the concentration of the amino acids arginine and proline was higher in the transgenic than in the isogenic line (t = 2.365, df = 13, p = 0.03 for arginine; t = 4.073, df = 13, p<0.001 for proline). There was no difference in total amino acid concentration within each pair.

**Figure 3:**
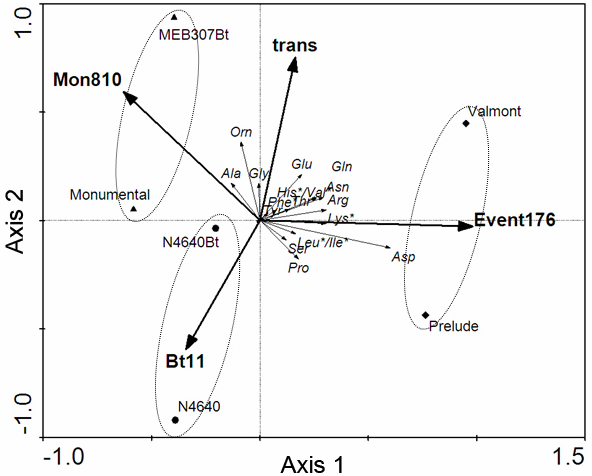
Correlating transformation with amino acid content. Distribution of amino acid concentrations (plotted as vectors) in samples of transgenic/isogenic pairs of maize plants belonging to three transformation events (vector Mon 810 denotes the pair MEB307Bt/Monumental; vector Bt11 denotes the pair N4640Bt/N4640; vector Event 176 denotes the pair Valmont/Prelude, vector trans indicates transgenic varieties belonging to all three transformation events) in the ordination biplot of an redundancy analysis (RDA). Axis 1 (Eigen Value = 0.054) and axis 2 (Eigen Value = 0.018) are presented. The ellipses group the different transgenic/isogenic pairs. For amino acid abbreviations see figure 2. Asterisks indicate the essential amino acids.

In order to explore underlying trends in the dataset, the distribution of the amino acid concentrations was investigated by redundancy analysis (RDA), a direct gradient analysis [25]. The RDA (Figure 3) indicates that relative ratios of investigated amino acids differ between transgenic and isogenic lines. Most amino acids (except proline, serine and leucine/isoleucine) were positively linked to the vector “trans” corresponding to the transgenic varieties, meaning that their concentrations tend to be higher in the transgenic lines. Moreover, different groups of amino acids correlate to different transgenic/isogenic pairs. Ornithine, alanine and glycine were linked to (higher in) the pair belonging to event Mon 810. The pair belonging to event 176 was positively correlated to almost all amino acids, whereas the pair belonging to event Bt11 was negatively correlated to most amino acids.

**Figure 4:**
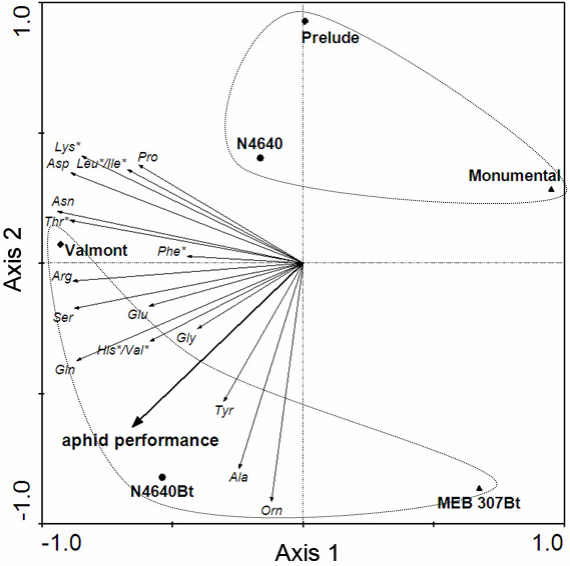
Amino acid content and aphid performance. Distribution of amino acid concentrations (plotted as vectors) in samples of transgenic/isogenic pairs of maize plants in the ordination biplot of a principal component analysis (PCA). Axis 1 (Eigen Value = 0.56) and axis 2 (Eigen Value = 0.19) are presented. The two clusters formed by either the transgenic or the isogenic varieties are indicated. The vector aphid performance indicates the colony performance of aphids (for details see figure 3). For amino acid abbreviations see figure 2. Asterisks indicate the essential amino acids.

To explore the relationship between aphid performance and amino acid concentration in the six maize varieties studies, we subjected the amino acid data to a principal component analysis (PCA). In the PCA of amino acid distribution two clusters were evident, one comprising the three isogenic lines and a second one comprising the three transgenic lines (Figure 4). The insertion of the vector for the colony performance of the aphids indicated that all amino acids are positively linked to aphid performance and show higher concentrations in the transgenic varieties. Glutamine and ornithine correlated best to performance and the high eigenvalues imply that these variables explain 75% of the variability. The coordinates of the varieties on this axis were consequently an adequate description of the amino acid data.

Although the above results indicate that a higher amino acid concentration in the transgenic lines may explain the enhanced aphid performance, a regression analysis between the coordinates and aphid performance gave a non-significant result (R^2^ = 0.51, F = 4.19, p = 0.11). The unavoidable small sample size (n = 6) calls for caution in the interpretation of this result.

### Effect of honeydew on *C. marginiventris* longevity and parasitism

**Figure 5:**
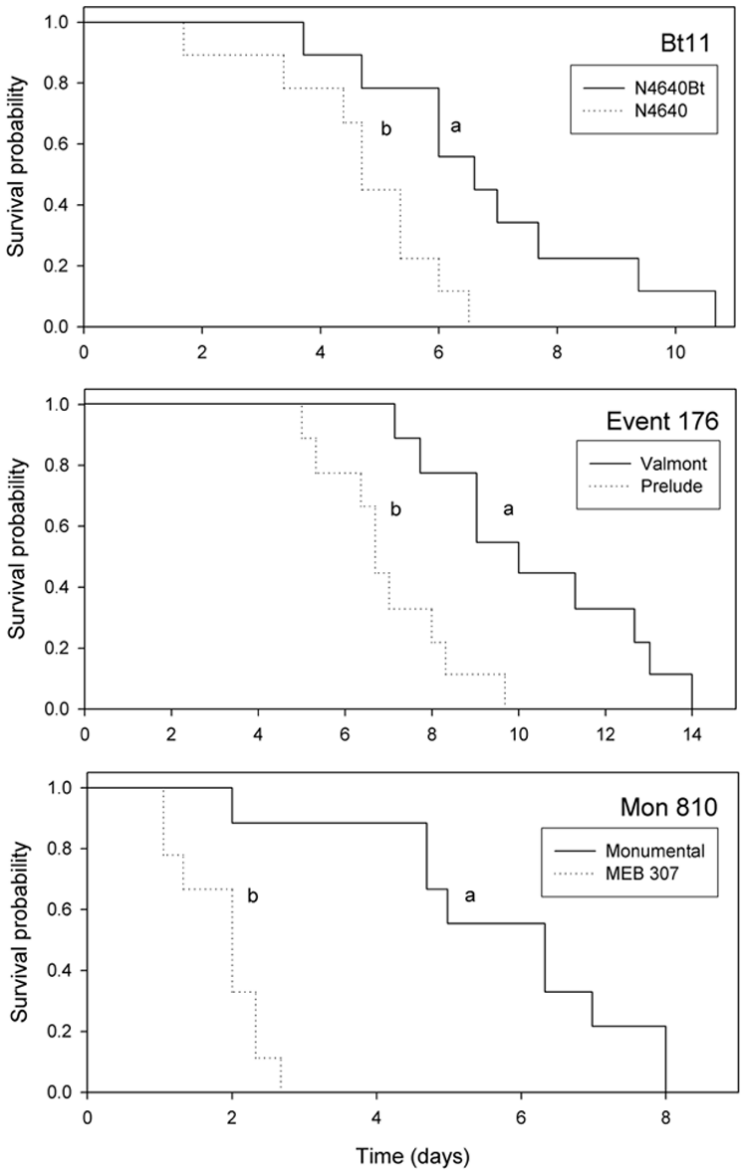
Parasitoid longevity. Survival curves showing the survival probability of *C. marginiventris* when feeding on honeydew produced by *R. maidis* on maize of transgenic/isogenic pairs belonging to three different events. Different letters indicate significant differences between curves (p<0.01).

Performance of *C. marginiventris* females was compared for wasps placed in cages with host-infested conventional maize next to aphid-infested maize of a transgenic or isogenic line. Survival and offspring production of the parasitoid differed between the transgenic and isogenic lines within each of the three events tested. *C. marginiventris* that fed on honeydew produced by aphids on the transgenic lines survived longer than the ones that fed on honeydew produced by aphids on the respective isogenic lines (for the pair N4640Bt/N4640 n = 9, χ^2^ = 7.3, df = 1, p = 0.006; for the pair MEB307Bt/Monumental n = 9, χ^2^ = 14.6, df = 1, p<0.001; for the pair Valmont/Prelude n = 9, χ^2^ = 9.9, df = 1, p = 0.001) (Figure 5).

**Figure 6:**
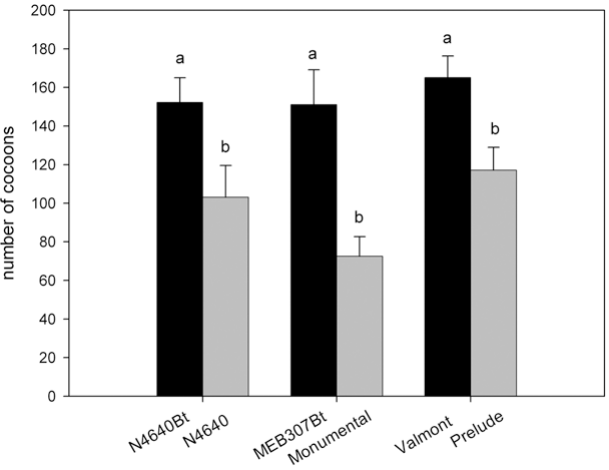
Parasitism. Total number of offspring (number of cocoons) produced by *C. marginiventris* when feeding on honeydew produced by *R. maidis* on different maize varieties. Different letters indicate significant differences between treatments within one transgenic/isogenic pair (p<0.05).

Similarly, parasitoid females that had fed on honeydew from *R. maidis* on transgenic plants produced more offspring (parasitized more hosts) than the females that had fed on honeydew from aphids on the respective isogenic lines (for the pair N4640Bt/N4640: t = 2.55, df = 16, p = 0.02; for the pair MEB 307Bt/Monumental: t = 3.79, df = 16, p = 0.002; for the pair Valmont/Prelude: t = 2.93, df = 16, p = 0.002) (Figure 6).

### Honeydew analysis

To test if differences in parasitoid performance were due to differences in the honeydew composition, we analysed the sugars in the honeydew from one transgenic and near isogenic pair of each of the three events. We also analysed the honeydew produced by *R. maidis* on other conventional maize varieties (Best, Byzance, Challenger, Delprim, Graf and Pactol) to access the overall variability in honeydew composition produced by *R. maidis* feeding on different maize genotypes.

**Figure 7:**
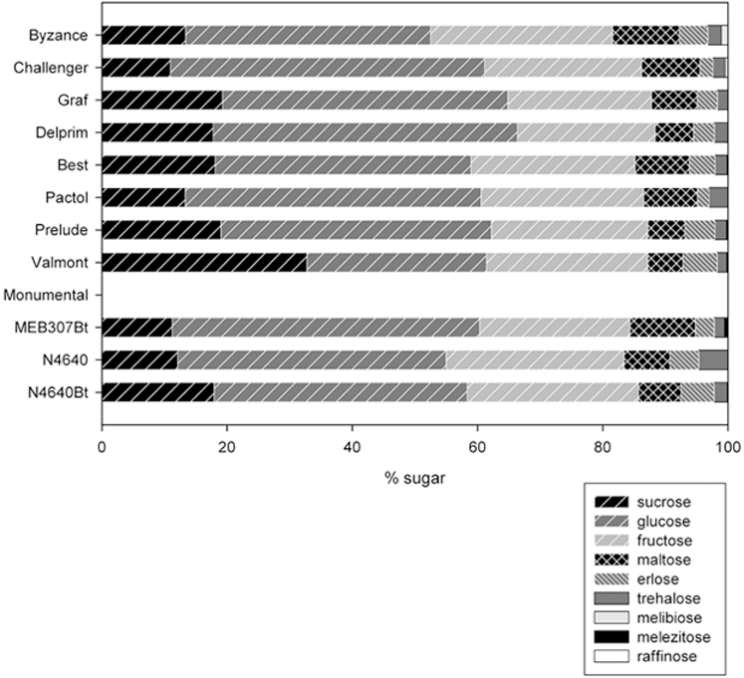
Sugar composition of the honeydew produced by the corn leaf aphid *R. maidis* feeding on different maize genotypes.

Sugar composition of the transgenic lines fell within the range of variation seen in conventional maize varieties, and the transgenic-isogenic pairs did not differ significantly for any of the sugars (separate t-tests on arcsine transformed proportions for each transgenic/isogenic pair, all p>0.05) (Figure 7). It was not possible to collect honeydew produced from the variety Monumental, as its high resistance to the aphid prevented colony establishment.

The typical phloem sugar sucrose and its hexose components, fructose and glucose, made up 81 to 88% of the sugars present in the honeydew produced on the varieties tested. The maltose found in the honeydew might be at least partially plant-derived as this sugar has been found in maize plants [27], [28]. Erlose was the most important aphid-synthesized sugar and trehalose was also present in all honeydews. Traces of melibiose were detected for the varieties N4640Bt, Challenger and Byzance; traces of melezitose were detected for N4640, N4640Bt, MEB 307Bt, Prelude, Valmont, Delprim; and traces of raffinose detected for Best, Challenger and Byzance.

### Correlation between honeydew intake and survival

For the wasps that were given a one time feeding bout on honeydew there was a positive correlation between intake and longevity of *C. marginiventris* (p<0.001) (for N4640Bt R^2^ = 0.56, for N4640 R^2^ = 0.60; for Valmont R^2^ = 0.47, for Prelude R^2^ = 0.50; for MEB 307Bt R^2^ = 0.60). The percentage weight gain of wasps that fed on honeydew produced by aphids on the respective transgenic and isogenic lines was not statistically different (t = 0.802, df = 38, p = 0.428 for N4640Bt/N4640; t = 1.037, df = 38, p = 0.306 for Valmont/Prelude) (average weight gain was 12.23% for N4640Bt, 10.90% for N4640; 12.24% for Valmont, 10.32% for Prelude; 11.65% for MEB 307Bt). Similarly, there was no difference in longevity between wasps feeding on honeydew from the transgenic and isogenic pairs (T = 391.5, p = 0.626 for N4640Bt/N4640; T = 1.037, p = 0.507 for Valmont/Prelude) (average longevity was 3.8 days for N4640Bt, 3.9 days for N4640; 3.7 days for Valmont, 3.6 days for Prelude; 3.7 days for MEB 307Bt). As the variety Monumental was resistant to *R. maidis* for the pair MEB307Bt/Monumental the results are only available for the transgenic line.

## Discussion

*R. maidis* colonies were found to perform considerably better on Bt maize than on the near isogenic correspondent lines. The only exception was for the Navaris/Antaris combination, both of which were found to be highly susceptible to the aphid, resulting in exceptionally high numbers on these plants (Figure 1). In this context, it should be pointed out that the performance of aphids on the studied Bt maize lines still falls well within the normal variation that is found among conventional maize lines. Enhanced performance on transgenic maize was reflected in the colony sizes, but was not measurable at the individual level. A similar discrepancy between individual and colony performance has been reported for the aphid *Cepegillettea betulaefoliae* [29]. This could explain why previous studies concluded that there is no difference in performance of aphids infesting Bt plants and their correspondent near isogenic lines [8], [30]. That aphids do indeed better on Bt maize also follows from the studies by Pons and colleagues, who found a significantly higher rate of offspring production by colonizing alate mothers of *R. padi* and consequently higher densities of this species on Bt maize [10], [11]. The observed differences in aphid numbers are unlikely only the result of a difference in attractiveness of the plants. In our experiments, the colonizing aphids were directly placed on their respective plants and did not have an option to move away. We rather think that the higher colony densities on Bt maize were caused by differences in chemical constituents that rendered the plants less well defended and/or more nutritious for the aphids (e.g. [31], [32]). Several studies have shown the key role of phloem amino acid concentration and composition in the phloem in determining aphid performance (e.g. [33]–[36]). However, this relationship is not always apparent [37], [38].

Although there were only weak statistical differences in amino acid composition between the lines, explorative data analyses (RDA) indicate significant correlations between different maize lines and the concentrations of individual amino acids. Transgenic lines in general had higher amino acid concentrations than the corresponding isogenic lines. This was especially true for the essential amino acids, which were all positively linked to the transgenic varieties with the exception of leucine/isoleucine (Figure 3). The PCA exploring the relationship between aphid performance and amino acid concentration also showed a positive, but marginally significant linkage. Hence, the differences in susceptibility levels between transgenic and isogenic varieties might partially be explained by the differences in the amino acid composition of the sieve element sap, but are unlikely to be the main explanation. Furthermore, each transgenic/isogenic pair had a quantitatively and qualitatively characteristic amino acid composition (Figure 3).

In addition to phloem amino acids, several other factors can influence the ability of an aphid species to exploit a host plant, such as physical characteristics [39]–[41] and secondary plant metabolites [42]–[45]. Indeed, one complimentary explanation for the higher susceptibility levels to aphids by the Bt plants is that, like in the maize varieties studied by Nie and colleagues [46], the Bt varieties used in our study might have lower levels of the hydroxamic acid DIMBOA (2,4-dihydroxy-7-methoxy-1,4-benzoxazin-3-one) and some phenolic acids compared to their non transgenic counterparts. These compounds have an important role providing resistance against herbivores of gramineous plants [47]. Other explanations, such as a difference in susceptibility that arose during breeding procedures after the transformation, cannot be ruled out [48], [49]. This is especially true for the dramatic difference in susceptibility to *R. maidis* between MEB 307Bt and Monumental. Indeed, although Monumental is the conventional line closest to MEB 307Bt, the two share only 97.5% genetic identity (information provided by Monsanto).

The higher numbers of *R. maidis* found on the Bt plants resulted in an overall increase of honeydew on the plants, which offered a significant advantage for *C. marginiventris* females that were allowed to feed on the honeydew (Video S1). The wasps lived significantly longer and produced more offspring (i.e. parasitized more host larvae on neighbouring plants) than females that had access only to aphid-infested near isogenic lines. Results from the additional two feeding experiments imply that this benefit was merely due to the increased honeydew quantity and not to a higher nutritional quality. In addition to the fact that more honeydew was present in the cages with transgenic plants, the aphid clusters on the isogenic plants were fewer and smaller, probably making it more difficult for the wasps to find them. Analyses of honeydew for sugar composition showed it to be similar for Bt maize and non-Bt maize. In order to cover their energetic needs, adult Hymenoptera mainly forage for sugars and have no additional need for nitrogen, which they ingested during the larval stages. Therefore, sugar composition is one of the key factors determining the nutritional value of honeydew [50]. The plant-derived sugars sucrose, glucose and fructose are of most value to insects, whereas the aphid-synthesized sugars may lower the nutritional value of honeydew [50], [51]. The analysis of the honeydew produced by *R. maidis* feeding on several maize varieties showed that only a small proportion of the sugars were aphid-produced. There were some differences in the composition of the honeydew produced by aphids feeding on the transgenic and the correspondent near isogenic lines, but these small differences fell well within the overall variability of the composition of the honeydew produced by *R. maidis* on conventional maize varieties (Figure 7). Moreover, in a previous study we found that in this system the aphid-produced sugars have no detectable effect on the quality of the honeydew as food for the parasitoid. [12].

That a difference in honeydew quality was not responsible for the observed increase in performance of wasps on Bt maize was also evident from the experiment in which females had a single feeding bout on different honeydews. They consumed comparable amounts of honeydew and after such a bout they survived just as well on honeydew from Bt maize as on honeydew from non-Bt maize, apparently honeydew palatability and quality were not affected by transformation of the maize lines. For optimal survival and reproduction, *C. marginiventris* needs to feed repeatedly on a sugar source [12]. Here we find that an increase in availability and accessibility of such a source facilitates this need.

It should not be assumed that for other plant species or other maize constructs, our results hold true. For instance, the potato aphid *Macrosiphum euphorbiae* has reduced growth and fecundity when reared on Cry3A potatoes, but it has an improved performance on transgenic potatoes producing rice cystatin I [52]. Furthermore, in other insects the Bt toxin may end up in the honeydew as is the case for the planthopper *Nilaparvata lugens* when it feeds on different varieties of transgenic rice containing different promoters, including CaMV 35S, the same promoter used in the events Mon 810 and Bt11 [53]. The planthopper shows no difference in performance on Bt and control lines, but they were found to produce more honeydew on Bt lines, and this honeydew was more acidic than the one from the control non-Bt lines [53]. Unlike for aphids, planthoppers feed on xylem and other non-phloem sources their honeydew is therefore a route of exposure of non-target organisms to Bt toxin.

For the maize lines studied here it can be concluded that increased susceptibility to aphids is advantageous to parasitoids that feed on aphid honeydew. This finding has important implications for the effectiveness of parasitoids as biological control agents; increased honeydew production not only helped to increase parasitoid longevity, but it also resulted in a significantly enhanced rate of parasitism. In maize monocultures, parasitoids usually have no direct access to plant-provided sources of sugar and aphids may be the only providers of these essential nutrients. In fact, aphid presence may, by being and/or producing an additional food source, help to sustain beneficial natural enemies of pest insects in a maize field. Future studies should determine, in the field, the exact implications of the higher susceptibility levels of Bt maize on aphid infestation levels and also on the performance of parasitoids and predators. Based on the current study it can be expected that as long as aphid numbers do not reach pest status, the unexpected and unintended increase of aphid susceptibility of Bt maize may pose an advantage in maintaining a beneficial insect fauna in Bt maize fields.

## Supporting Information

Movie S1 *Cotesia marginventris* feeding on aphid honeydew
(9.35 MB MOV)
